# Conservation of Zebrafish MicroRNA-145 and Its Role during Neural Crest Cell Development

**DOI:** 10.3390/genes12071023

**Published:** 2021-06-30

**Authors:** Tomás J. Steeman, Juan A. Rubiolo, Laura E. Sánchez, Nora B. Calcaterra, Andrea M. J. Weiner

**Affiliations:** 1 Instituto de Biología Molecular y Celular de Rosario (IBR), Consejo Nacional de Investigaciones Científicas y Técnicas (CONICET)—Facultad de Ciencias Bioquímicas y Farmacéuticas, Universidad Nacional de Rosario (UNR), Ocampo y Esmeralda, Rosario S2000EZP, Argentina; steeman@ibr-conicet.gov.ar (T.J.S.); calcaterra@ibr-conicet.gov.ar (N.B.C.); 2 Departamento de Zoología Genética y Antropología Física, Facultad de Veterinaria, Universidade de Santiago de Compostela, Campus de Lugo, 27002 Lugo, Spain; ja.rubiolo@usc.es (J.A.R.); lauraelena.sanchez@usc.es (L.E.S.)

**Keywords:** microRNA, neural crest, gene regulatory network, embryonic development

## Abstract

The neural crest is a multipotent cell population that develops from the dorsal neural fold of vertebrate embryos in order to migrate extensively and differentiate into a variety of tissues. A number of gene regulatory networks coordinating neural crest cell specification and differentiation have been extensively studied to date. Although several publications suggest a common role for microRNA-145 (miR-145) in molecular reprogramming for cell cycle regulation and/or cellular differentiation, little is known about its role during in vivo cranial neural crest development. By modifying miR-145 levels in zebrafish embryos, abnormal craniofacial development and aberrant pigmentation phenotypes were detected. By whole-mount in situ hybridization, changes in expression patterns of *col2a1a* and Sry-related HMG box (Sox) transcription factors *sox9a* and *sox9b* were observed in overexpressed miR-145 embryos. In agreement, zebrafish *sox9b* expression was downregulated by miR-145 overexpression. In silico and in vivo analysis of the *sox9b* 3′UTR revealed a conserved potential miR-145 binding site likely involved in its post-transcriptional regulation. Based on these findings, we speculate that miR-145 participates in the gene regulatory network governing zebrafish chondrocyte differentiation by controlling *sox9b* expression.

## 1. Introduction

The neural crest (NC) is a transient, multipotent stem cell-like population whose formation occurs early in development at the border of the neural tube. After closure of the neural tube, NC cells (NCCs) experience an epithelial-to-mesenchymal transition in order to delaminate and migrate away to some of the most distant positions of any embryonic cell type [1]. NCCs differentiate into a variety of derivatives, including neurons and glia of the enteric, sensory, and autonomic nervous system, pigment cells, chromaffin cells, bone and cartilage of the face, endocrine cells, cardiac structures, smooth muscle cells, and tendons [2]. Several well-characterized gene regulatory networks (GRNs) govern NC development, which when disrupted can lead to various neurocristopathies such as craniofronto-nasal dysplasia, DiGeorge syndrome, and certain forms of cancer [3,4]. Some actors critical for cellular regulation of gene expression are microRNAs (miRNAs), one type of small endogenous noncoding RNAs important in post-transcriptional gene silencing. MiRNAs bind to target mRNAs and regulate protein expression by repressing translation and/or promoting degradation of the target mRNA at the post-transcriptional level through the RNA-induced silencing complex [5]. MiRNAs comprise 1–2% of all genes in animals, and since each miRNA is predicted to regulate hundreds of targets, it is thought that half of protein-coding genes are under their control [5]. MiRNA’s biological roles are widely diverse, becoming active players in developmental embryogenesis, cell differentiation, organogenesis, growth, and programmed cell death, as well as stem and germ cell maintenance, disease, and evolution [6].

The process of NC diversification initiates with the activation of differentiation pathways in subpopulations of migratory cells [4]. These pathways operate under a positive feed-forward loop where initial NC regulators function together with locally activated differentiation effector genes [4,7]. In order to arrive at the proper target region, NCCs have to interpret multiple environmental signals that directly influence the place to which they migrate to settle and differentiate. The Sry-related HMG box (Sox) transcription factors Sox9 and Sox10 are major players regulating effector genes that provide specific characteristics to cells. While Sox9 is first expressed during NC specification in premigratory and early delaminating cells, Sox10 is detected in delaminating and migrating NCCs [1,7]. Sox9 also plays an essential role in cartilage development and chondrocyte differentiation at multiple steps; it regulates the expression of the Col2a1 gene, which encodes the major extracellular matrix protein, type-II collagen, in gnathostome vertebrate cartilage [8,9]. SOX9 defects in humans lead to campomelic dysplasia characterized by major defects in cartilage and bone [10]. Although much is understood about how SOX9 regulates cartilage matrix synthesis and hence joint function, less is known about the mechanisms controlling its own expression [11,12]. Some evidence suggests that miR-145 targets and suppresses the expression of Sox9, inhibiting chondrogenic differentiation of murine embryonic mesenchymal cells, as well as critically affecting human articular chondrocyte function [13,14]. As a result, miR-145 has been used as a metric to qualitatively assess the efficacy of this differentiation process [15]. On the other hand, miR-145 has been involved in other processes mainly related to cancer and embryonic stem cell differentiation [16,17]. Regarding cancer, mir-145 was reported to act as a tumor suppressor and has been shown to be downregulated in several cancer types [18,19,20,21,22,23,24,25]. Consequently, miR-145 was proposed as a candidate biomarker for cancer diagnosis, monitoring, and prognosis in humans [17]. In human embryonic stem cells, increased levels of miR-145 inhibit self-renewal, repress the expression of pluripotency genes, and induce lineage-restricted differentiation [16].

During zebrafish development, miR-145 is strongly expressed in gut smooth muscle and regulates its formation [26]. In addition, miR-145 is expressed in vascular and visceral smooth muscle cells and promotes their differentiation [27]. Other data uncover a cascade of molecular events where miR-145, together with other factors, governs lateral plate mesoderm differentiation and intestinal smooth muscle cell development in zebrafish [28]. Another study in zebrafish revealed that miR-145 regulates embryonic liver size by controlling hepatocyte proliferation. In this process, miR-145 directly targets progranulin A-dependent hepatic outgrowth during embryonic development [29]. In addition, miR-145 and several regeneration-associated factors have been involved in an early panretinal induction of octamer-binding transcription factor 4, which is essential for Müller glia reprogramming and cell cycle exit in zebrafish [30]. Finally, miR-145 and other miRNAs assist Müller glial cells of the zebrafish retina in reprogramming and forming progenitors essential for regeneration upon injury [31]. Altogether, data collected during zebrafish development suggest a common role for miR-145 in molecular reprogramming for cell cycle regulation and/or cellular differentiation, mainly over NC derivatives [32]. Supporting this notion, it has been shown that metformin, a popular antidiabetic drug, interferes with NCC determination by deregulating the canonical Wnt axis and a set of miRNAs, among which is miR-145 [33]. When murine embryonic stem cells are differentiated into NCCs in the presence of metformin, miR-200c and miR-145 show more than 5-fold upregulation over other miRNAs. Studies in different mouse and human cultured cells confirmed that Sox9 is a direct target of miR-145 [13,14,33,34], though the regulation of this pathway during embryonic development has not been assessed yet. In the present study, we analyzed in vivo the role of miR-145 during embryonic development, focusing on its action upon NC formation and differentiation using zebrafish as an animal model. Functional experiments indicate that miR-145 is involved in proper pigment cell differentiation, craniofacial development, cellular apoptosis, and expression patterns of cranial NC genes, likely by impairing the post-transcriptional regulation of *sox9b* during zebrafish NC development. The strikingly high degree of miR-145 conservation throughout evolution suggests that its role in mammalian cell culture is conserved in zebrafish development.

## 2. Materials and Methods

### 2.1. Zebrafish Care

All zebrafish were handled according to relevant national and international guidelines. All procedures using zebrafish from the Calcaterra lab were authorized by the Comité Institucional para el Cuidado y Uso de Animales de Laboratorio of the Facultad de Cs. Bioquímicas y Farmacéuticas-Universidad Nacional de Rosario, which has been accepted by the Ministerio de Salud de la Nación Argentina (files N° 6060/374 and 207/2018). Adult zebrafish were maintained at 28 °C on a 14:10 h light:dark cycle as previously described [35]. Matings usually involved crossing three males and four females in the same spawning tank. For mutant embryos, one female and one male were set up in crossing tanks. All embryos were staged according to morphological development in hours or days post-fertilization (hpf or dpf, respectively) at 28 °C [36]. Except for the CRISPR experiment (Section 2.3), embryos were injected at the one-cell stage into the yolk immediately below the cell using a gas-driven microinjection apparatus (MPPI-2 Pressure Injector, Applied scientific Instrumentation; Eugene, OR, USA).

### 2.2. In Silico Analysis of miR-145 Gene and Targets

Mature and stem-loop sequences of mir-143 (30 species) and miR-145 (25 species) were obtained from miRbase release 22 [37] and aligned with the Clustal Omega tool [38]. Loci and flanking regions (human: chromosome 5: 149,406,689–149,432,835 [+], GRCh38:CM000667.2; zebrafish: chromosome 14: 38,743,941–38,744,053 [+], GRCz11:CM002898.2) were obtained from Ensembl release 99 [39]. Synteny between human and zebrafish miR-145 was analyzed by comparing adjacent genes in terms of chromosome conservation. Putative targets for zebrafish miR-145 were obtained from TargetScanFish release 6.2 [40] and analyzed for Gene Ontology [41,42] and KEGG pathway [43] enrichment with DAVID [44], PANTHER [45], and g:Profiler [46]. RNA folding prediction was performed with RNAfold [47] and NUPACK [48].

### 2.3. MiR-145 Over- and Downregulation

For the overexpression experiments, the genomic region of zebrafish miR-145 (chromosome 14: 38,743,647–38,744,217) was amplified and cloned into a pSP64T-dsRED vector, using *Eco*RI-*Xho*I restriction sites (primer sequences in Supplementary Table S1, restriction sites in lowercase). To produce mRNA for microinjection, the plasmid was linearized with *Bam*HI (Invitrogen, Carlsbad, CA, USA) and transcribed with mMESSAGE mMACHINE^®^ SP6 (Invitrogen), alongside a no-miRNA control-dsRED vector. One-cell embryos were microinjected with 1.25 ng of the transcripts and incubated at 28 °C until collection.

Pure commercial 1-1Dimethylbiguanide hydrochloride (metformin; Sigma-Aldrich, St. Loius, MO, USA FG:165.62) was used for all the incubation experiments at LC_50_ = 7.5 mM concentration [49]. Dilutions were carried out using autoclaved reverse osmosis water. Embryos were obtained and allowed to develop until the 512-cell stage in osmosis water, then they were selected and incubated in the presence of metformin.

For the generation of CRISPR mutants, target selection and sgRNA design were performed using the browser extensible data track [50], “CRISPRs” track on the ZebrafishGenomics track hub) and CRISPRScan [51]. To disrupt the miR-145 gene, a pair of gRNAs (100 ng each, Supplementary Table S1) targeting the miRNA sequence were prepared by PCR and in vitro transcription [52], and then mixed with 5 μg of Cas9 protein (Truecut, Invitrogen). The gRNA complexes were mixed with the Cas9 protein and incubated for 10 min at 37 °C to form the ribonucleoprotein (RNP) complex. The RNP complexes were injected (3 nL) into the animal pole of one-cell stage embryos.

### 2.4. Zebrafish DNA Extraction, Genotyping, and Characterization of the CRISPR/Cas9-Induced Mutation

Adult fish were anesthetized with 0.04% tricaine methanesulfonate (MS222). PCR-ready genomic DNA was isolated from whole zebrafish embryos (24 hpf) or tail fin clips of adult zebrafish. Briefly, tissue samples were incubated in a thermal cycler with 70 µL of extraction buffer (10 mM Tris pH8, 50 mM KCl, 1.5 mM MgCl_2_, 0.3% (*v*/*v*) Tween20, 0.3% *v*/*v* NP-40) at 94 °C for 20 min, then 2.5 µL of 20 mg/mL Proteinase K were added and further incubated at 55 °C for 1h, ending with 94 °C for 20 min. For genotyping, PCR reactions (20 μL total volume) were prepared using Taq Pegasus (PB-L, Buenos Aires, Argentina) according to the manufacturer’s instructions (primer sequences in Supplementary Table S1) and visualized with 3% (*w*/*v*) agarose gel electrophoresis stained with Gel Green (Biotium, Freemont, CA, USA). To characterize the knockout mutation, DNA samples were analyzed by Sanger sequencing.

### 2.5. RT-qPCR

RNA from 35 embryos was obtained with TRIzol^®^ reagent according to the manufacturer’s instructions (Invitrogen) and treated with DNAse RQ1 (Promega, Madison, WI, USA). Retrotranscription was performed with Mu-MLV RT enzyme (Promega) and oligo(dT) primers. Quantitative PCR was done with Taq Platinum as per the manufacturer’s instructions (Invitrogen), using SYBR Green (Sigma-Aldrich), on an Eppendorf RealPlex4 thermocycler. For miRNA detection, specific stem–loop primers were added to the retrotranscription reaction [53]. *Rpl13* and *ef1α* were used as endogenous controls [54]. All primer sequences are detailed in Supplementary Table S1. Data analysis was performed with qBase software v2.2 and statistical analysis with GraphPad Prism 7, following MIQE guidelines [55].

### 2.6. Pigment Quantification 

In order to observe the reflecting iridophores, 35 larvae of 72 hpf were photographed with an Olympus MVX10 stereoscopic microscope and Olympus C-60 ZOOM digital camera, over a dark background and with 45° incident light. Reflection intensity was measured with Quantifish software [56] and statistical analyses were performed with GraphPad Prism 7.

### 2.7. Alcian Blue staining

To analyze the effect on cranial structures, 40 larvae of 5 dpf were fixed with 4% (*w*/*v*) paraformaldehyde in PBT 1X (PBS 1X with 0.1% (*v*/*v*) Tween-20) overnight at 4 °C, washed 4 times with PBT 1X, and stained as detailed elsewhere [57]. Pictures were taken with an Olympus MVX10 stereoscopic microscope and Olympus C-60 ZOOM digital camera. The different cranial cartilage parameters were measured as reported elsewhere [4] with ImageJ software (National Institute of Health, Bethesda, MD, USA) [58].

### 2.8. Acridine Orange Staining

Twenty-four hpf embryos were manually dechorionated and stained with Acridine Orange to detect apoptotic cells as reported elsewhere [59]. Fifty fluorescent embryos were photographed with an Olympus MVX10 stereoscopic microscope and Olympus C-60 ZOOM digital camera. Fluorescence levels were quantified with QuantiFish software [56] and analyzed with GraphPad Prism 7.

### 2.9. Whole-Mount In Situ Hybridization (WISH)

Embryos were staged as previously described, manually dechorionated (for embryos under 72hpf), fixed with 4% (*w*/*v*) paraformaldehyde in PBT 1X overnight at 4 °C, washed 3 times with PBT 1X, and stored in methanol at −20 °C. The staining protocol was performed as detailed elsewhere [60]. Riboprobes were labeled with Digoxigenin-UTP according to the manufacturer’s instructions (Roche Diagnostics, Mannheim, Germany). Embryos were imaged with an Olympus MVX10 stereoscopic microscope and Olympus C-60 ZOOM digital camera.

### 2.10. Reporter Assays

The d4EGFPn-3′UTR reporters were constructed using specific primers for the *sox9b* 3′UTR genomic region (primer sequences in Supplementary Table S1, restriction sites in lowercase). Two different fragments (1 and 2) were separately amplified, purified, and cloned into a pGEM-T Easy Vector System (Promega). Then, both fragments were separately sub-cloned at the *Xho*I site of the pSP64-T-d4EGFPn vector, and the direction of insertion confirmed by sequencing. Positive plasmids for each construction were cut with *SmaI* and used as a template for transcription with mMESSAGE mMACHINE^®^ SP6 according to the manufacturer’s instructions (Invitrogen). Empty pSP64-T-d4EGFPn was used as a control. Transcripts were microinjected into one-cell embryos at 1.5 ng together with 1.25 ng of either miR-145-dsRED or control-dsRED transcripts. D4EGFPn fluorescence was observed at the 50 epiboly stage. Embryos were imaged with a Leica MZ16F stereoscopic microscope and a Nikon DS-Fi1 digital camera. Fluorescent levels were examined with QuantiFish software [56] and statistical analyses were performed with GraphPad Prism 7.

## 3. Results

### 3.1. MiR-145 Is Conserved throughout Evolution

**Figure 1:**
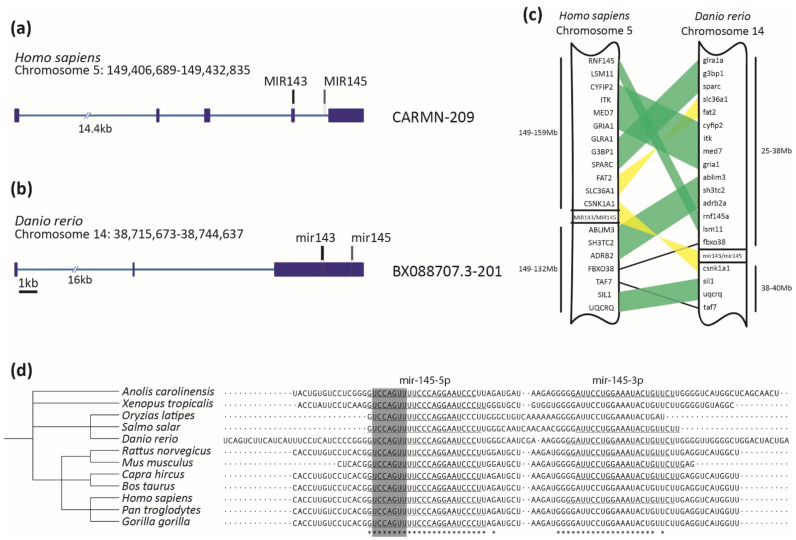
MiR-145 is highly conserved across vertebrates. (**a**,**b**) *Homo sapiens CARMN-209* and *Danio rerio* BX088707.3-201 transcripts containing miR-143 (in black) and miR-145 (in gray) precursors. Exons are represented with dark blue boxes and introns with light blue lines. Scale bar represents 1kb. (**c**) Schematic representation of synteny analysis of miR-143/miR-145 cluster between *Homo* *sapiens* and *Danio rerio.* Blocks of genes in the same order are highlighted in green, while those blocks that are inverted are in yellow. Only conserved genes are shown. (**d**) Clustal Omega alignment of miR-145 precursor, displaying taxonomic tree on the left and aligned sequences on the right. Asterisks at the bottom represent 100% conserved bases. The mature miR-145-5p sequence is underlined on the left, with the seed sequence highlighted in gray. Some species that also possess annotated mature miR-145-3p are underlined on the right.

Zebrafish miR-145 is a single copy gene, as was detected for human and other vertebrates. Importantly, the miR-145 sequence has not been found in nonvertebrate genomes, suggesting a specific clade function. To infer miR-145 gene structure, both human and zebrafish pri-miR-145 were searched for in the Ensembl database in the corresponding genomes. Zebrafish miR-145 is located on chromosome 14 (GRCz11 14: 38,743,941–38,744,053 [+]) while human miR-145 is located on chromosome 5 (GRCh38 5: 149,430,646–149,430,733 [+]) (Figure 1a,b). In both species, miR-145 is organized in long noncoding RNAs (*CARMN* in humans and BX088707.3 in zebrafish, Figure 1a,b), demonstrating a structural genomic conservation; nevertheless, the lncRNAs themselves show low sequence conservation (46.62% identity). The search for the miR-145 gene sequence led to the identification of an evolutionarily conserved miR-143–145 cluster both in zebrafish and humans (Figure 1a–c), showing a structural genomic conservation among vertebrates, which is in agreement with previous information [61]. Although some degree of shuffling was observed, gene synteny analysis revealed preservation of several blocks ranging from two to four genes upstream of the miR-145 locus and in the same order between the two species. Downstream of the miR-145 locus, the order of syntenic genes was less preserved, although several genes were still found to be conserved between chromosomes of the two species (Figure 1c). Therefore, in addition to the conservation of the gene structure, the genomic contexts have also been conserved in the two species. In order to explore conservation of pre-miR-145 and pre-miR-143 sequences, a broader group of species was compared. First, pre-miR sequences from mammalian, amphibian, and teleost fish species were collected from miRbase and their pairwise identities determined with Clustal Omega, with identity percentages ranging from 61.54% to 100% (average 90%) for miR-145 and from 71.29% to 100% (average 92%) for miR-143 (Figure 1d, Supplementary Figures S1 and S2, Supplementary Tables S2 and S3). Data showed that miR-145 and miR-143 mature sequences are remarkably conserved among vertebrates. Furthermore, the seed region, a key element in target recognition and translation inhibition, is 100% conserved in both miRNAs, suggesting that not only the targets but also the functions of the two miRNAs have been conserved among vertebrates. 

**Figure 2:**
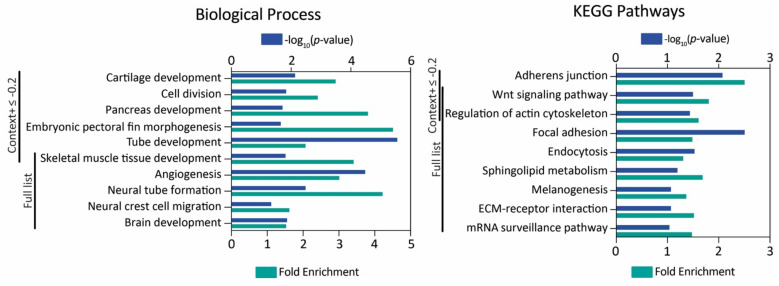
MiR-145 zebrafish target gene analysis. Gene Ontology and KEGG pathway enrichment analysis of genes with context+ score under −0.2 and the full list. The -log_10_(*p*-value) and fold enrichment of selected biological process terms (**left**) and KEGG pathways (**right**) are shown.

Both in humans and zebrafish, the miR-145-5p strand is the form predominantly detected (miRBase). Therefore, from now onwards, we refer to this form as “miR-145”, unless stated otherwise. A list of 2997 putative miR-145 target zebrafish genes identified by means of TargetScan (Supplementary Table S4) was analyzed for Gene Ontology and KEGG pathway enrichment. Firstly, we analyzed those genes with a context+ score under −0.2, and then we explored the full list (Figure 2, Supplementary Table S5). A total of 23 and 34 biological process terms were found to be over-represented with DAVID (*p*-value < 0.05, FDR test) for the short and full gene list, respectively. Likewise, 12 and 59 terms were found with PANTHER (*p*-value < 0.05, FDR test). With g:Profiler, the full list was analyzed, since the order of the query list was taken into consideration, and 190 terms were found (*p*_adj_ < 0.05, g:SCS threshold, ordered query as context+ score). Regarding KEGG pathways, four (short list) and 15 (full list) pathways were detected as over-represented with DAVID (*p*-value < 0.1, FDR test), and 27 with g:Profiler (*p*_adj_ < 0.05, g:SCS threshold, ordered query as context+ score). Focusing on embryonic development, multiple terms related to the NC and its derivatives were found to be statistically enriched.

### 3.2. Expression Analysis of miR-145 during Zebrafish Development

**Figure 3:**
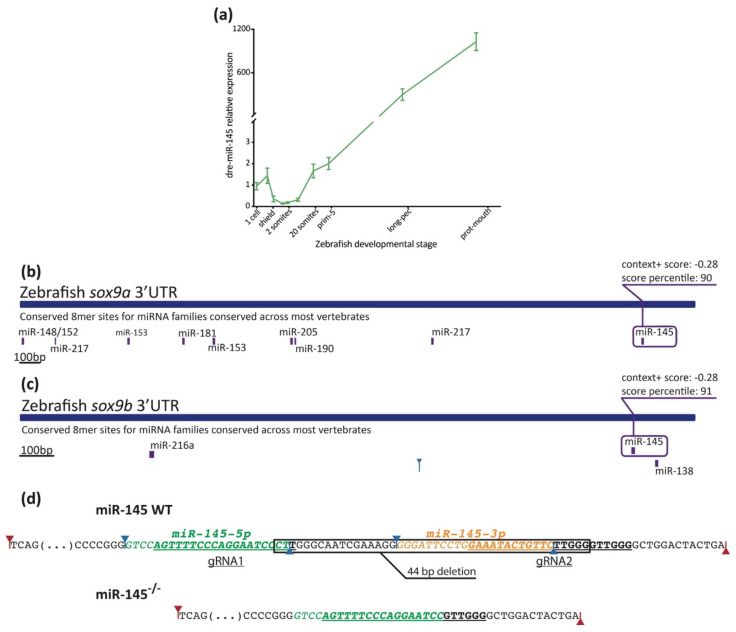
Measurement of miR-145 expression with stem–loop RT-qPCR. (**a**) MiR-145 temporal expression levels during zebrafish development (±SEM, *n* = 3). (**b**,**c**) Schematic representation of *sox9a* (ENSDARG00000003293) and *sox9b* (ENSDARG00000043293) 3′UTR. Putative miRNA binding sites are shown below each region. MiR-145 sites are enclosed in purple boxes, with their respective context+ scores. (**d**) Schematic representation of miR-145 genomic wild-type sequence (**top**) and CRISPR/Cas9 mutant sequence (**bottom**). Mature mir-145-5p and -3p in green and orange, respectively, and underlined gRNA sequences are shown. Drosha and Dicer1 cleavage sites marked with red and blue arrows, respectively.

The zebrafish miR-145 developmental expression pattern has already been characterized [26,62]. From 16 to 24 hpf, miR-145 is ubiquitously expressed at very low levels. Between 24 and 72 hpf, its expression increases specifically in the heart, ear, and pharyngeal arches [26,62]. At 96 hpf, miR-145 is localized in both the gut and swim bladder, being stronger within the gut smooth muscle layer [26,62]. As there were no expression records regarding the early developmental stages, we analyzed miR-145 expression levels from the one-cell stage onwards by performing stem–loop RT-qPCR. Results showed that miR-145 is maternally inherited and expressed at high levels during the first stages of zebrafish embryonic development (Figure 3a). Over the course of development, miR-145 expression decreases, reaching a minimum around the bud and 10-somite stages. From that stage onwards, miR-145 expression increases, with the highest expression measured at 3 dpf.

A negative correlation is observed when comparing miR-145 (Figure 3a) and *sox9b* expression behavior (reported in Dooley et al., 2019 [11]). Indeed, among one to eight somites, the expression of *sox9b* is higher and miR-145 is lower. Later on, between 20 somites and prim-25, the expression of *sox9b* is low while the expression of miR-145 increases. Both *sox9a* and *sox9b* 3′UTRs have putative target binding sites for miR-145 (Figure 3b,c; TargetScan, score: −0.28), and both transcription factors are included in several developmental process GO terms (Figure 2). These observations suggest a role for miR-145 in both *sox9a* and *sox9b* expression regulation during zebrafish development.

### 3.3. Effects of miR-145 Over- and Downregulation on Neural Crest Derivatives

In order to study the role of miR-145 during development, we performed gain- and loss-of-function experiments in zebrafish embryos. We microinjected a pri-miRNA in tandem with a reporter dsRED sequence to increase the miR-145 levels (miR-145-dsRED). As a control, an mRNA generated from the construct that contained only the dsRED sequence was injected in the same conditions (control-dsRED). During embryonic development, we assessed both the presence of reporter protein by fluorescence and the pri-miRNA processing by stem–loop RT-qPCR. An increase in miR-145 levels in microinjected wild-type embryos was observed at 24 hpf (Supplementary Figure S3A). As metformin upregulates miR-200c and miR-145 while murine embryonic stem cells are differentiated into NCCs [33], we tested whether miR-145 levels increased in metformin-treated embryos. Surprisingly, metformin-treated embryos showed a reduction in the levels of miR-145 at 24 hpf (Supplementary Figure S3A). For all our experiments, 7.5 mM metformin was used, according to the concentration established by FET-test (LC_50_ = 7.5 mM) [49]. This metformin concentration is lower than that used in other experiments performed in zebrafish embryos [33] and could explain the differences observed. For specific loss-of-function experiments, we generated a CRISPR knockout mutant with a deletion of 44 bp in the miR-145 stem–loop region (Figure 3d and Supplementary Figure S3B). The CRISPR deletion eliminated the last two nucleotides of miR-145-5p (CT) and the complete sequence of miR-145-3p (Figure 3d). Even though the sequence of miR-145-5p is virtually intact, predictions suggest that a stem–loop structure could potentially be formed, but Dicer1 processing would result in a shorter miR-145-5p, since part of its sequence would be included in the new loop of the stem–loop structure (Supplementary Figure S3C). Heterozygous miR-145^+/−^ developed normally, being phenotypically indistinguishable from wild-type siblings; embryos were raised to adulthood, genotyped for the deletion, and crossed to generate homozygous mutants. In homozygous miR-145^−/−^ mutants, initial development appeared morphologically normal up to 5 dpf. Homozygous miR-145^−/−^ mutants were phenotypically indistinguishable from wild-type siblings; embryos were raised to adulthood, genotyped for the deletion, and crossed to generate homozygous mutants. By stem–loop RT-qPCR, the expression level of miR-145 declined up to 96% in miR-145^−/−^ mutant embryos at 24 hpf (Supplementary Figure S3A), suggesting that pri-miRNA processing did not happen. Therefore, in our experimental conditions, miR-145 overexpression was reached by microinjecting a pri-miRNA in tandem with a reporter dsRED sequence, while miR-145 downregulation was indirectly achieved by incubating developing zebrafish embryos in the presence of metformin and directly by using the miR-145^−/−^ zebrafish knockout mutant.

#### 3.3.1. Effects on Pigmentation

**Figure 4:**
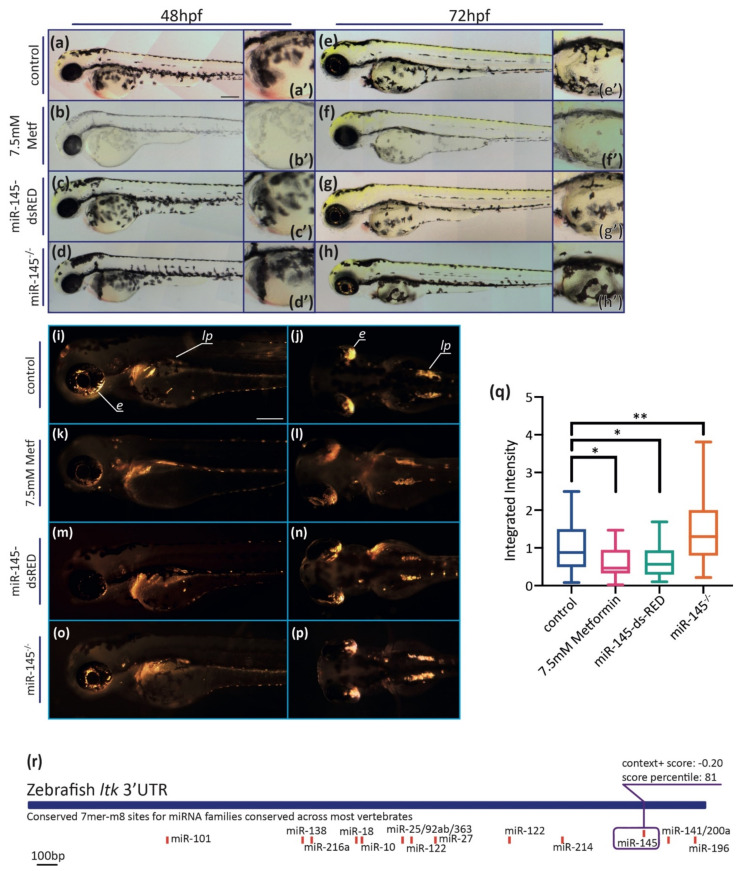
MiR-145 gain- and loss-of-function larvae display pigment developmental phenotypes. (**a**–**h**) Representative images of live metformin-treated (**b**,**f**) or miR-145-dsRED microinjected (**c**,**g**), and miR-145^−/−^ mutant (**d**,**h**) larvae and control siblings (**a**,**e**). Lateral views with head to the left, dorsal uppermost, of 48 hpf (**a**–**d**) and 72 hpf (**e**–**h**) specimens. Insets show enlarged regions of the yolk sac (**a′**–**h′**) in order to compare melanocyte differentiation phenotype. Scale bar represents 200 μm in A for a-h. (**i**–**p**) Incident light images of 3 dpf larvae showing eye and trunk iridophores (silver spots) in wild-type sibling (**i**,**j**), and metformin-treated (**k**,**l**), miR-145-dsRED microinjected (**m**,**n**), and miR-145^−/−^ mutant (**o**,**p**) larvae. Pictures in (**i**,**k**,**m**,**o**) are lateral views while pictures in (**j**,**l**,**n**,**p**) are dorsal views, head to the left. Scale bar represents 200 μm in (**i**) for (**i**–**p**). (**q**) Quantification of iridescent light of miR-145-dsRED microinjected, metformin-treated, and miR-145^−/−^ mutant larvae with their corresponding controls at 3 dpf. *e*: eyes; *lp*: lateral patches. (±SEM, *n* = 3, two-tailed Student’s *t*-test, * *p* < 0.05; ** *p* < 0.01). (**r**): Schematic representation of *ltk* (ENSDARG00000042681) 3′UTR. Putative miRNA binding sites are shown below each region. MiR-145 site is enclosed in a purple box, with its respective context+ score.

Zebrafish pigmentation is characterized by the presence of melanocytes, iridophores, and xanthophores, three different kinds of pigment cells derived from NCCs [63]. Melanocytes showing the black pigment melanin are evident by 25 hpf onwards, initiating in the region just posterior to the otic vesicle [63]. From 28–32 hpf, metformin-treated embryos showed a reduced number of pigmented melanocytes, as previously reported [33], especially in the posterior hindbrain region (not shown). By 48 hpf, metformin-treated embryos displayed a decreased number of melanocytes, as well as decreased melanin levels (visible as grayer, less black, coloration) in the remaining melanocytes (Figure 4b). At 72 hpf, differences in the pattern of melanocytes located in the head, lateral stripe, and yolk were observed in metformin-treated larvae (Figure 4f). No detectable phenotypic defects in melanocyte differentiation were observed in embryos and larvae microinjected with the miR-145-dsRED construct nor in miR-145^−/−^ zebrafish mutants (Figure 4c,d,g,h). Importantly, black pigmentation of the pigmented retinal epithelium (where melanocytes derive from the brain) was unaffected in metformin-treated fish (Figure 4b,f), suggesting a specific effect of metformin on black pigment cells derived from the NC.

Iridophores produce the iridescent pigment in the eyes, on the top of the head, scattered amongst melanocytes in the body, and in discrete ‘lateral patches’ above the yolk sac in the anterior trunk, as seen in 72 hpf control larvae (Figure 4i,j). Phenotypic analysis in both miR-145-dsRED microinjected and metformin-treated larvae showed a decrease in the extent of iridophores on the eye and in the lateral patches (Figure 4k–n). On the contrary, an increase in the iridophore pattern was detected in miR-145^−/−^ mutant larvae (Figure 4o,p). When quantifying the iridescent pigment, lower levels were detected in miR-145-dsRED microinjected and metformin-treated larvae, but higher levels in miR-145^−/−^ mutants when compared with controls (Figure 4q). Putative miR-145 binding sites were searched in silico in genes belonging to the zebrafish iridophore GRN [64]. The analysis showed the presence of a miR-145 target binding site in *ltk* 3′UTR (the receptor tyrosine kinase leukocyte tyrosine kinase; Figure 4r). These results revealed an unidentified role for miR-145 in iridophore development that will need deeper exploration.

#### 3.3.2. Effects on Craniofacial Development

**Figure 5:**
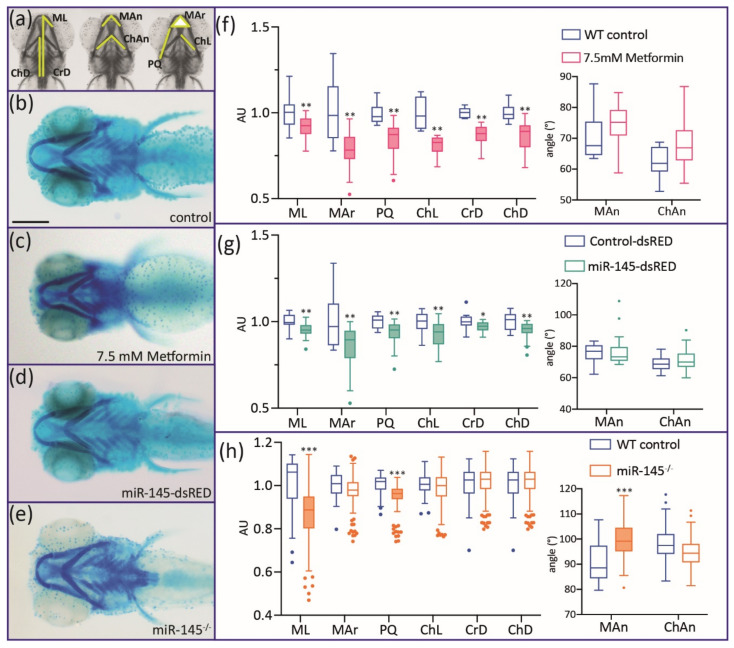
Alcian Blue staining of 5 dpf miR-145-dsRED microinjected, miR-145^−/−^ mutant, and metformin-treated larvae. (**a**) Different cranial parameters are measured: Mar: Meckel area; ChL: ceratohyal length; PQ: palatoquadrate length; ML: Meckel length; CrD: cranial distance; ChD: ceratohyal-to-fin distance; Man: Meckel angle; ChAn: ceratohyal angle. (**b**–**e**) Control (**b**), metformin-treated (**c**), miR-145-dsRED microinjected (**d**), and miR-145^−/−^ mutant (**e**) larvae. All larvae are positioned ventrally with head to the left. (**f**–**h**) Measurement of cranial parameters corresponding to metformin-treated (**f**), miR-145-dsRED microinjected (**g**), and miR-145^−/−^ mutant (**h**) larvae compared with their respective controls (WT siblings not treated with drug, Control-dsRED microinjected, and WT siblings, respectively). Arbitrary units (AU) in the *y*-axis of length and area graphs result from standardization of measurement data to the control counterpart of each parameter. Statistically different parameters have boxplots shaded for easier identification. Two-tailed Student’s *t*-test, * *p* < 0.05, ** *p* < 0.01, *** *p* < 0.0001. Scale bar represents 200 μm in (**b**) for (**b**–**e**).

Defects in craniofacial development were clearly detected when embryos microinjected with miR-145-dsRED were fixed at 5 dpf and stained with Alcian Blue (Figure 5d, see A for definition of measured parameters). MiR-145 overexpression caused significant shortening in the lengths of Meckel (ML), ceratohyal (ChL), and hyosymplectic–palatoquadrate (PQ) cartilages (Figure 5g; *N* = 30). The Meckel area (MAr, defined as the triangle shaped by Meckel cartilages), the distance between ceratohyal cartilage joints and the lateral fins, and the cranial distance (measured as the distance from the anterior-most M to the lateral fins) also showed significant reductions (compare Figure 5b with Figure 5d). However, the angles formed by the Meckel and ceratohyal cartilages were not significantly modified (Figure 5g; *N* = 30). For miR-145^−/−^ mutant larvae, a milder phenotype was observed, detecting only significant reductions in ML and PQ lengths and for Meckel angle (Figure 5e,h). In the case of metformin-treated larvae, more severe phenotypes were detected, showing significant reductions in all cartilage areas and lengths (Figure 5c,f; LC_50_ metformin concentration). In vivo results suggest a role for miR-145 during zebrafish craniofacial development, in line with previous records gathered in mouse and human cell chondrogenesis processes studied in cultured cells [13,14]. 

The analysis of all the results obtained so far revealed that metformin treatment broadly affects pigment cell and craniofacial NC derivatives whereas specific miR-145 over- and downregulation seem to affect iridophore and craniofacial structure development. The phenotype observed for iridophore development is striking and should be studied further. Previously reported results for zebrafish [33], along with the data described here, suggest that metformin treatment could not only alter miR-145 expression and melanocyte differentiation, but also affect other developmental processes by apparently additional complex unrelated activities of the drug. As a result, we decided to not test metformin-treated embryos any further. 

### 3.4. MiR-145 Over- and Downregulation Induces Apoptosis during Zebrafish Development

**Figure 6:**
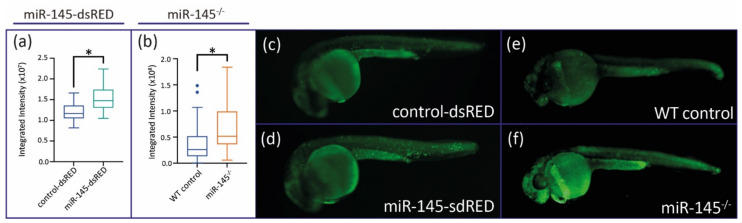
Apoptosis levels are analyzed in miR-145 gain- and loss-of-function embryos. (**a**) Quantification of Acridine Orange fluorescence of control-dsRED and miR-145-dsRED microinjected embryos at 24 hpf. (**b**) Quantification of Acridine Orange fluorescence of WT control and miR-145^−/−^ mutant embryos at 24 hpf. Student’s *t*-test, * *p* < 0.0001. (**c**–**f**) Pictures of control-dsRED (**c**), miR-145 overexpressing (**d**), WT (**e**), and miR-145^−/−^ (**f**) 24 hpf embryos stained with Acridine Orange.

In zebrafish organogenesis, miR-145 regulates liver size by controlling hepatocyte proliferation [29]. As noted above, both miR-145-dsRED microinjected and miR-145^−/−^ mutant fish show an apparent decrease in the size of the head and craniofacial cartilages. We wondered whether the smaller head size was due to increased apoptosis of cephalic-most cells. To test this, we used Acridine Orange staining of apoptotic cells to assess in vivo cell death in control and treated embryos. At 24 hpf, miR-145-dsRED microinjected embryos displayed significantly higher levels of apoptosis in the whole embryo when compared with control-dsRED microinjected embryos (Figure 6a,b; *N* = 50). MiR-145^−/−^ mutant embryos also show significantly higher levels of apoptosis at 24 hpf when compared with WT controls (Figure 6c; *N* = 50). The increased apoptosis in treated embryos could explain the phenotypes affecting NC derivatives. 

### 3.5. MiR-145 Overexpression Affects the Expression of NCC Marker Genes

**Figure 7:**
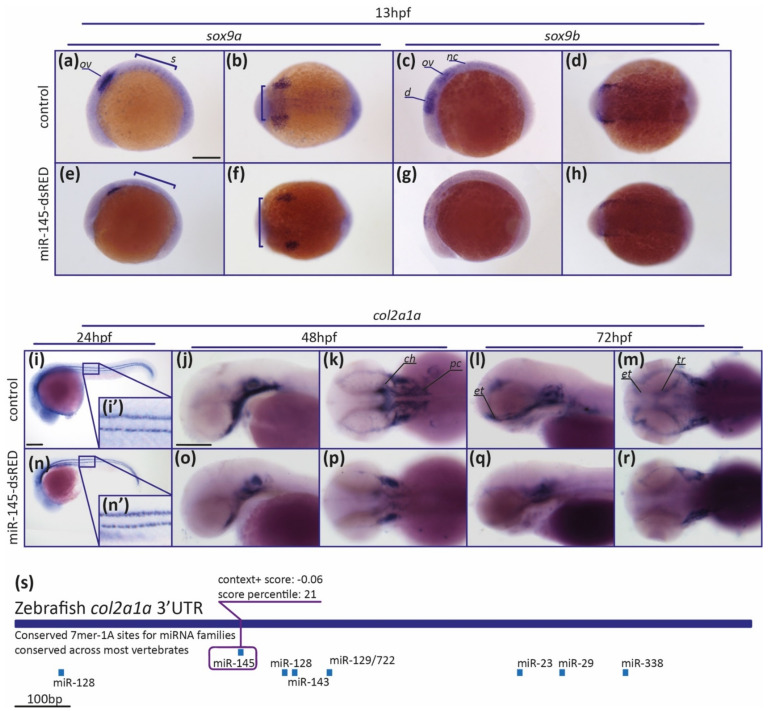
MiR-145 upregulation affects the expression of NCC marker genes. (**a**–**h**) Whole-mount in situ hybridization of *sox9a* (**a**,**b**,**e**,**f**) and *sox9b* (**c**,**d**,**g**,**h**) performed on 13 hpf miR-145-dsRED microinjected embryos. Lateral views are dorsal to top (**a**,**e**,**c**,**g**); dorsal views are anterior to left (**b**,**f**,**d**,**h**). In (**a**) ov: otic vesicle; s: somites. In (**c**) d: diencephalon; ov: otic vesicle; nc: neural crest. Scale bar represents 200 μm in (**a**) for (**a**–**h**). (**i**–**r**) Whole-mount in situ hybridization of *col2a1a* (ENSDARG00000069093) in miR-145-dsRED microinjected specimens at 24 hpf (**i**,**n**), 48 hpf (**j**,**k**,**o**,**p**), and 72 hpf (**l**,**m**,**q**,**r**). Lateral and dorsal views are head to left. In (**k**) ch: ceratohyal; pc: parachordals. In (**l**,**m**) et: ethmoid plate; tr: trabeculae. (**s**) Schematic representation of *col2a1a* 3′UTR. Putative miRNA binding sites are shown below each region. MiR-145 site is enclosed in a purple box, with its respective context+ score. Scale bars represent 200 μm in (**i**) for (**i**,**n**); and in (**j**) for (**j**–**m**) and (**o**–**r**).

To deepen the study of miR-145’s role in craniofacial development, we analyzed by whole-mount in situ hybridization (WISH) the *sox9a/b* and *col2a1a* gene expression patterns in specimens microinjected with miR-145-dsRED. At 13 hpf, control embryos expressed *sox9a* in NCCs of the otic vesicle and the somites (Figure 7a,b; regions labeled with ov and s, respectively). MiR-145-dsRED overexpressing embryos consistently showed a diminution of *sox9a* expression in the somites, when compared with controls (black brackets in Figure 7a,e (85% affected, *N* = 26)). When analyzing *sox9a* expression from the dorsal view, an altered pattern in NC patches was observed (brackets in Figure 7b,f (85% affected, *N* = 26)). At 13 hpf, control embryos expressed *sox9b* in NCCs of the diencephalic, midbrain, and hindbrain regions (Figure 7c,d; regions labeled with d, m, and h, respectively). A consistent reduction in *sox9b* expression territory in the diencephalic, midbrain, and hindbrain regions was detected in miR-145-dsRED microinjected embryos at 13 hpf (Figure 7c,d,g,h (78% affected, *N* = 40)). In conclusion, data indicate that miR-145 overexpression affects both the *sox9a* and *sox9b* positive staining territories, suggesting a regulatory role for miR-145 in their mRNA expression patterns.

*Col2a1a* expression is required for chondrocyte differentiation [65]. At 24 hpf, embryos express *col2a1a* along the notochord vacuolar cells (Figure 7i). No differences in *col2a1a* expression were detected in miR-145-dsRED overexpressing embryos (Figure 7n). In control 48 hpf specimens, *col2a1a* expression was detected in presumptive craniofacial structures and the notochord. At this stage, cartilage begins to differentiate morphologically, and it is possible to visualize early pre-cartilage condensations in the developing parachordals, which form the neurocranium together with the ethmoid plate and trabeculae, and the ceratohyal and the notochord (Figure 7j,k). In miR-145-dsRED overexpressing embryos, a clear diminution of *col2a1* expression in the parachordals and ceratohyal was detected (Figure 7o,p (75% affected, *N* = 32)). At 72 hpf, *col2a1a* expression was detected in the presumptive neurocranium, pectoral fins, and pharyngeal skeleton of wild-type larvae (Figure 7l,m). Regarding miR-145-dsRED overexpressing larvae, *col2a1a* expression defects were detected in the parachordals, ethmoid plate, and trabeculae, while *col2a1a* expression in the developing pharyngeal skeleton was less altered (Figure 7q,r (88% affected, *N* = 34)). A putative miR-145 binding site was detected in silico in the *col2a1a* 3′UTR (Figure 7s), suggesting a direct regulatory role for miR-145 over Col2a1a expression during chondrocyte differentiation. These results confirmed the abnormal development of craniofacial structures when miR-145 levels are modulated and allow us to speculate that the facial cartilage formation is dependent on normal miR-145 expression during zebrafish embryonic development.

The expression patterns of *myod1*, *egr2b*, and *tbxta* in both miR-145-dsRED and control-dsRED microinjected embryos were similar, thus ruling out possible nonspecific effects of the treatment (Supplementary Figure S4). 

### 3.6. The Expression of sox9b Can Be Regulated at the 3′UTR Level

Evidence gathered in different *in cellulo* experimental approaches suggests that miR-145 targets and suppresses Sox9 expression [13,14,33,34], in agreement with the in silico and in vivo results gathered in this work (Figure 3c and Figure 7g–l). 

**Figure 8:**
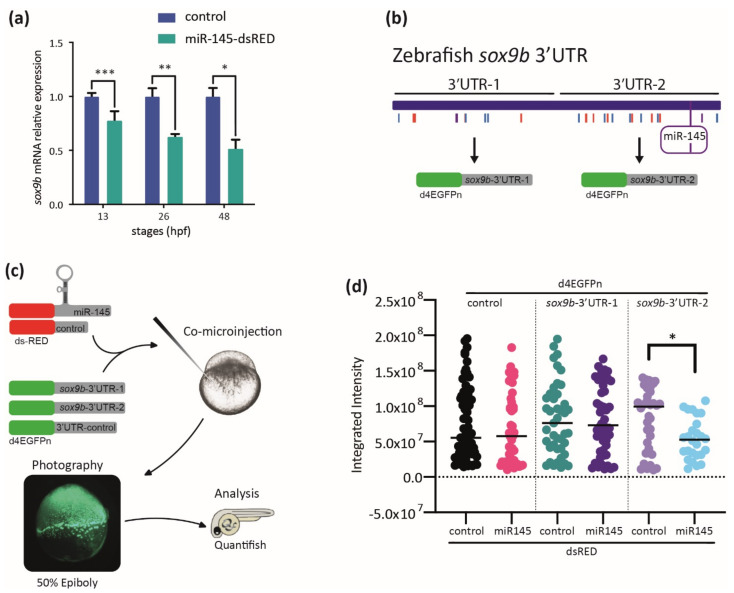
MiR-145 controls sox9b expression. (**a**) Relative levels of *sox9b* mRNA in miR-145-dsRED microinjected and control embryos; bars represent values measured at each developmental stage normalized to the amount of the corresponding mRNA measured in controls at 13, 26, 48, and 72 hpf (±S.E.M., *n* = 4). Two-tailed Student’s *t*-test, * *p* = 0.03; ** *p* = 0.012; *** *p* = 0.002. (**b**) Schematic representation of the cloning of *sox9b* 3′UTR fragments 1 and 2, with putative miRNA sites marked with colored lines and miR-145 enclosed in a purple box. (**c**) Schematic representation of the experimental strategy followed for the study of dGFP expression. (**d**) Quantification of dGFP in embryos co-microinjected with miR-145-dsRED or control-dsRED and different 3′UTRs (*n* = 4). Two-tailed Student’s *t*-test, * *p* = 0.006.

To further study the consequences of miR-145 overexpression, we evaluated *sox9b* expression during zebrafish development. The relative amount of *sox9b* mRNA measured by RT-qPCR assays was significantly lower in miR-145-dsRED microinjected embryos at all developmental stages analyzed (Figure 8a). To test the potential role of the miR-145 binding site identified in silico (Figure 3c) in this regulation, the *sox9b*-3′UTR sequence was cloned in two fragments downstream of a destabilized GFP coding sequence (d4EGFPn, named dGFP). The dGFP-*sox9b*-3′UTR-1 (*sox9b*-3′UTR-1; sequence in exon 3, from −2 bases to +1035 bases from the stop codon’s last base) contains the first 1000 bp and no putative miR-145 binding sites while the dGFP-*sox9b*-3′UTR-2 (*sox9b*-3′UTR-2; sequence in exon 3, from +1073 to +2014 bases from the stop codon) contains the second 1000 bp and a putative miR-145 binding site (Figure 8b). A third chimera fusing the dGFP-coding region to the SV40-poly(A) sequence was constructed to test as a control. The three constructs (*sox9b*-3′UTR-1, *sox9b*-3′UTR-2, and control) were used to synthesize in vitro their corresponding mRNAs, which were independently co-injected with either miR-145-dsRED or control-dsRED mRNAs in one-cell zebrafish embryos (Figure 8c). Injected embryos were allowed to develop until 50 epiboly and both the dGFP and dsRED fluorescences were assessed under the microscope; the dGFP expression was measured by green fluorescent quantification while red fluorescence was used to normalize the signal in each assay (Figure 8d). The presence of either miR-145-dsRED or control-dsRED mRNAs did not affect the levels of dGFP synthesized from control or *sox9b*-3′UTR-1 sequences. However, the co-injection of *sox9b*-3′UTR-2 mRNA with miR-145-dsRED led to a significant reduction in the levels of green fluorescence when compared with fluorescence measured in embryos co-injected with control-dsRED mRNA. Both *sox9b*-3′UTR-1 and *sox9b*-3′UTR-2 have been analyzed out of the endogenous genomic context; therefore, the existence of an artificial mRNA decay cannot be ruled out. However, the finding that the decrease in fluorescence relative intensity was detected only in the case of *sox9b*-3′UTR-2 suggests that any part of this fragment contains elements potentially involved in miR-145-mediated *sox9b* expression regulation. Our data suggest that, in developmental stages wherein NC migration and differentiation are taking place, *sox9b* is post-transcriptionally regulated by miR-145.

## 4. Discussion

Several studies have revealed a role for miRNAs as essential modulators of development and disease. Mir-145 is a highly conserved single-copy small RNA that is co-transcribed with miR-143 as a cluster [66]. Our in silico analysis uncovered that miR-145 structural and functional features have been conserved throughout evolution (Figure 1). Mir-145 pri-miR sequences show high conservation between mammalian, amphibian, and teleost fish species; also, human and zebrafish miR-145 genomic organization and context have been conserved. The absence of miR-145 sequences in the genomes of nonvertebrates suggests an evolutionary gain in vertebrates and allows us to speculate about a potential function of miR-145 in biological processes unique to vertebrates. The NC and placodes are two notable exceptions in vertebrates that do not derive from populations of evolutionarily ancient cells (e.g., mesoderm and endoderm). Both NCCs and placodes are responsible for constructing many of the traits that uniquely define the clade, including the cartilage and bone of the head and jaw skeleton, neurons and glia of the peripheral sensory nervous system, colorful patterns of pigmentation, and much more [67]. A complex and organized set of genetic interactions and intercellular signaling pathways define the regulatory state of NCCs from their earliest stages in the neural plate border to their differentiation. Further studies are required to determine how miR-145 is spatially and temporally involved in the NC regulatory pathways during vertebrate development.

Here, we found that miR-145 is maternally inherited, thus raising the possibility of playing a larger role in maternal mRNAs that is not fully understood yet. Additionally, we confirmed deficiencies in melanocyte development as a consequence of metformin treatment (Figure 4 and [33]). In melan-a cells (a mouse cell line with normal pigment production), miR-145 overexpression or downregulation led to reduced or increased expression of *Sox9*, *Mitf*, *Tyr*, *Trp1*, *Myo5a*, *Rab27a*, and *Fscn1* [68]. Human melanocytes transfected with miR-145 displayed perinuclear accumulation of melanosomes with additional hypopigmentation of harvested cell pellets [68]. However, specific miR-145 over- or misexpression in developing zebrafish did not produce detectable changes in melanocytes, suggesting a different regulatory role for miR-145 throughout zebrafish melanocyte GRN players. The differences could also be explained by taking into account that melan-a are cultured cells, while our experiments were carried out in a complex multicellular organism. Of note, effects on iridophores had not been reported before (Figure 4). Based on a combination of genetic experimentation and mathematical modeling, the GRN associated with iridophore specification and differentiation has been considerably expanded and refined in embryonic zebrafish [64]. Recently, we have found a decrease in the extent of iridophores in the eye and in the lateral patches of zebrafish *dicer1^sa9205^* homozygotes and dicer1 morphants [55]. Here, we explore the hypothesis that iridophore GRN could be modulated through the activity of miR-145 and possibly with other miRNAs, reaching the proper cell specification and differentiation. In particular, in silico analysis suggests a putative binding site for miR-145 in the 3′UTR of *ltk*, a key player for iridophore differentiation. 

Studies performed using cultured cells revealed that miR-145 is involved in regulating critical cartilage extracellular matrix genes (COL2A1 and aggrecan) and tissue-specific miRNAs (miR-675 and miR-140), being proposed as an important regulator of human chondrocyte function and a new target for cartilage repair [13]. *In cellulo* works have linked miR-145 with cell viability and osteogenic differentiation [69,70,71,72]. In addition, experiments performed in conditional *Dicer* mutant mice (knockout Cre driven by *Pax2* promoter) indirectly suggest that miR-145 plays a role during palatal development [73]. It has been shown that secondary palatal development in mice becomes morphologically arrested prior to mineralization with a significant decrease in the expression levels of miR-101b, miR-140, and miR-145. Nonetheless, in vivo craniofacial defects due to miR-145 up- or downregulation had not been previously reported. In zebrafish, cranial NCCs from the anterior midbrain give rise to the medial ethmoid plate; cranial NCCs at the midbrain–hindbrain boundary forms the trabecular rods and lateral ethmoid plate; slightly more posterior cranial NCCs are involved in the pterygoid process of the palatoquadrate; while even more posterior cranial NCCs contribute to the Meckel’s cartilage and palatoquadrate [74,75,76]. Modulation of miR-145 induced changes in all the cranial NCC derivatives, suggesting that proper levels of miRNA are required at early NC developmental stages (Figure 5). Several mechanisms may explain the abnormal NC development observed in zebrafish embryos over- or misexpressing miR-145. Acridine Orange assays show that high levels of miR-145 increase apoptosis of zebrafish embryonic cells, suggesting that NCCs may die before differentiation (Figure 6). This finding agrees with data gathered in embryonic and cancer cultured cell lines, wherein miR-145 overexpression promotes apoptosis and reduces cell viability [16,34,69,77,78]. These observations as a whole could suggest that miR-145 participates in the control of cell death regulation in both normal and pathological conditions. 

Apart from the role of miR-145 in NC precursor survival, the modulation of miR-145 levels may also hamper gene regulatory control of key NC markers. In vitro evidence regarding the existence of Sox9 epigenetic regulation by miR-145 during cell differentiation has been reported [13,14,34]; however, scant evidence of such regulation has been gathered in vivo to date. Experiments carried out in this work revealed different defects in the early somitogenesis stage caused by aberrant *sox9a* and *sox9b* gene expression patterns due to miR-145 upregulation (Figure 7). Results suggest that miR-145 function is not essential for cranial NC migration in zebrafish, but it collaborates in determining the extent of the *sox9a*-expressing cell population. Indeed, miR-145 overexpression leads to strong downregulation of *sox9b* expression during development. In addition, our in vivo assay shows that the presence of miR-145 affects the amount of dGFP for *sox9b*-3′UTR-2 and suggests that *sox9b* expression is regulated by miR-145. *In cellulo* studies stated that miR-145 represses Sox9 protein translation [13,14,34]. Here, we detected that miR-145-dependent regulation of *sox9b* expression occurs at both mRNA stabilization (Figure 7k,l and Figure 8a) and protein synthesis levels (Figure 8d) in developing zebrafish embryos. Therefore, the aberrant *col2a1a* expression detected in miR-145-overexpressing larvae could be due to a direct regulation by miR-145 over the *col2a1a* 3′UTR, and/or be a consequence of *sox9b* downregulation, and/or synergy of both effects. 

The novelty of our work lies in the finding that miR-145 participates in zebrafish NC development, thus opening new opportunities to address questions regarding the role of miRNAs in NCC differentiation during embryonic development. It is noteworthy that, when overexpressing miR-145, fewer silver iridescent cells were observed, while in miR-145 knockout mutant fish, larger amounts of iridophores were detected. The craniofacial structures were smaller by either over- or down-expressing miR-145 when compared with controls. All these results reinforce the notion that miR-145 is involved in chondrocyte differentiation and craniofacial development, as well as supporting the use of zebrafish as a valuable tool for disease modeling.

## Data Availability

Not applicable.

## Supplementary Materials

The following are available online at https://www.mdpi.com/article/10.3390/genes12071023/s1, Supplementary Figure S1: Conservation of miR-143 across vertebrate species. Pre-miR-143 sequence alignment of 30 vertebrate species using Clustal Omega, showing taxonomic tree on the left and hairpin sequences on the right. Asterisks represent 100% conserved bases and the seed sequences for miR143-5p and miR143-3p are enclosed in gray rectangles. Supplementary Figure S2: Conservation of miR-145 across vertebrate species. Pre-miR-145 sequence alignment of 25 vertebrate species using Clustal Omega, showing taxonomic tree on the left and hairpin sequences on the right. Asterisks represent 100% conserved bases and the seed sequences for miR145-5p are enclosed in gray rectangles. Supplementary Figure S3: Measurement of miR-145 levels in 24 hpf treated embryos. A: Effects of metformin treatment, miR-145-dsRED microinjection and miR-145^−/−^ mutant on miR-145 expression levels. (±SEM, *n* = 3, two-tailed Student’s *t*-test, * *p* = 0.0026, ** *p* = 0.0006, *** *p* < 0.0001). B: miR-145^−/−^ CRISPR/Cas9 genotyping. Left: 3% agarose gel stained with Gel Green. MWM: Molecular weight marker, Ladder50bp. Right: Representation of pre-miR-145 (purple), deleted fragment (red) and genotyping primers (green). C: RNA folding predictions of WT (left) and mutant (right) pre-miR-145 sequences, with their minimal free energy (MFE) scores. Supplementary Figure S4: Effects of miR-145 in the expression of different marker genes. Whole-mount in situ hybridization of myoD (A,B,F,G), krox20 (C,H), and ntl (D,I) performed on 13 hpf miR-145-dsRED or control-dsRED microinjected embryos. Lateral views are dorsal to right (A,C,D,F,H,I); dorsal views are anterior to top (B,G). Whole-mount in situ hybridization of ntl in miR-145-dsRED (J) or control-dsRED (E) microinjected specimens at 24 hpf, lateral views are head to left. Scale bars represent 200 μm in A for A–D and F–I; and in E for E and J. Supplementary Table S1: Primer sequences for cloning and RT-qPCR. Supplementary Table S2: Sequence identity matrix of pre-miR-143 of 30 vertebrate species. Supplementary Table S3: Sequence identity matrix of pre-miR-145 of 25 vertebrate species. Supplementary Table S4: List of zebrafish genes extracted from TargetScan.org and used for Gene Ontology analyses with context+ score. Supplementary Table S5: Gene Ontology results.
