# Supplementary material for: Conservation of Zebrafish MicroRNA-145 and Its Role during Neural Crest Cell Development
Source: Genes (Basel). 2021 Jun 30;12(7):1023. doi: 10.3390/genes12071023 (PMC8306979; doi:10.3390/genes12071023)
Supplement: Supplementary file 1 [file genes-12-01023-s001.zip › genes-1242264-supplementary-figures.pdf]

genes

MDPI

Article

Conservation of Zebrafish MicroRNA-145 and its Role during Neural Crest Cell Development.

Tomas J Steeman [1], Juan A Rubiolo [2], Laura E Sánchez [2], Nora B Calcaterra [1] and Andrea MJ Weiner [1,*]

Supplementary Materials

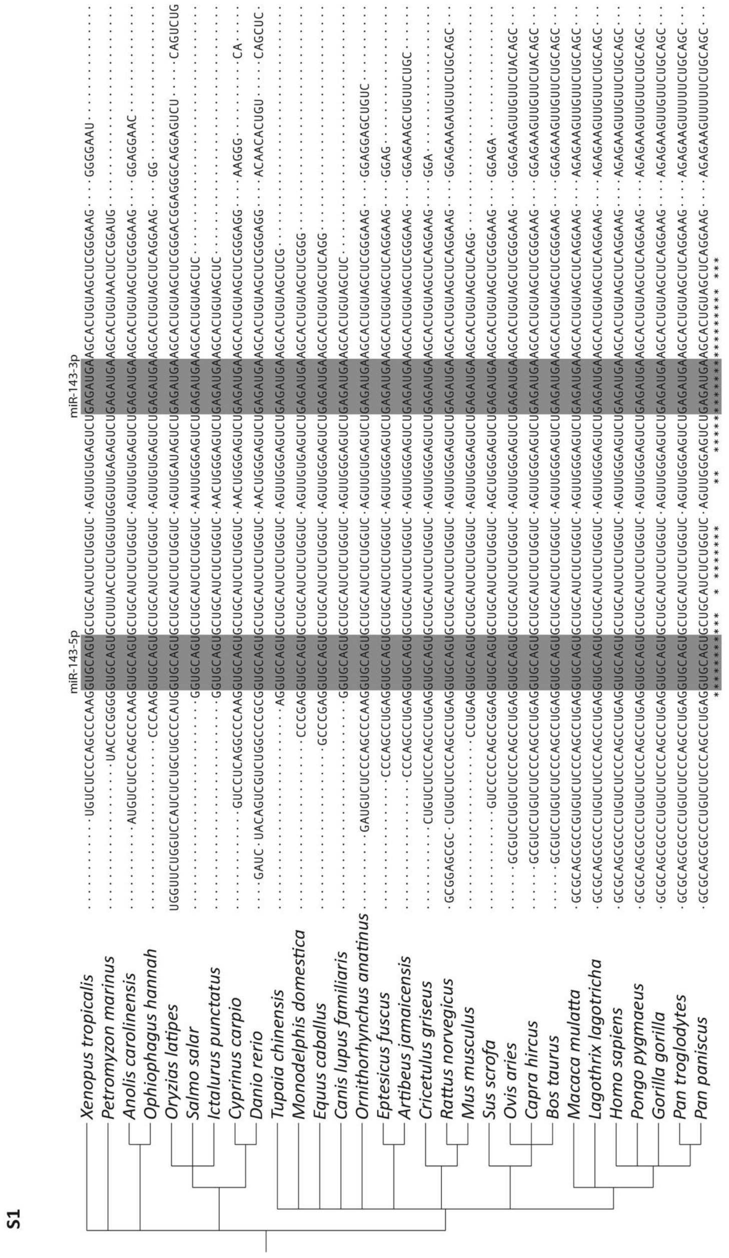

Figure S1. *Conservation of miR-143 across vertebrate species.* Sequence alignment of pre-miR-143 of 30 vertebrate species using Clustal Omega, with taxonomic tree on the left and hairpin sequence on the right. Asterisks represent 100% conserved bases and enclosed in gray rectangles are the seed sequences for miR143-5p and miR143-3p.

S2

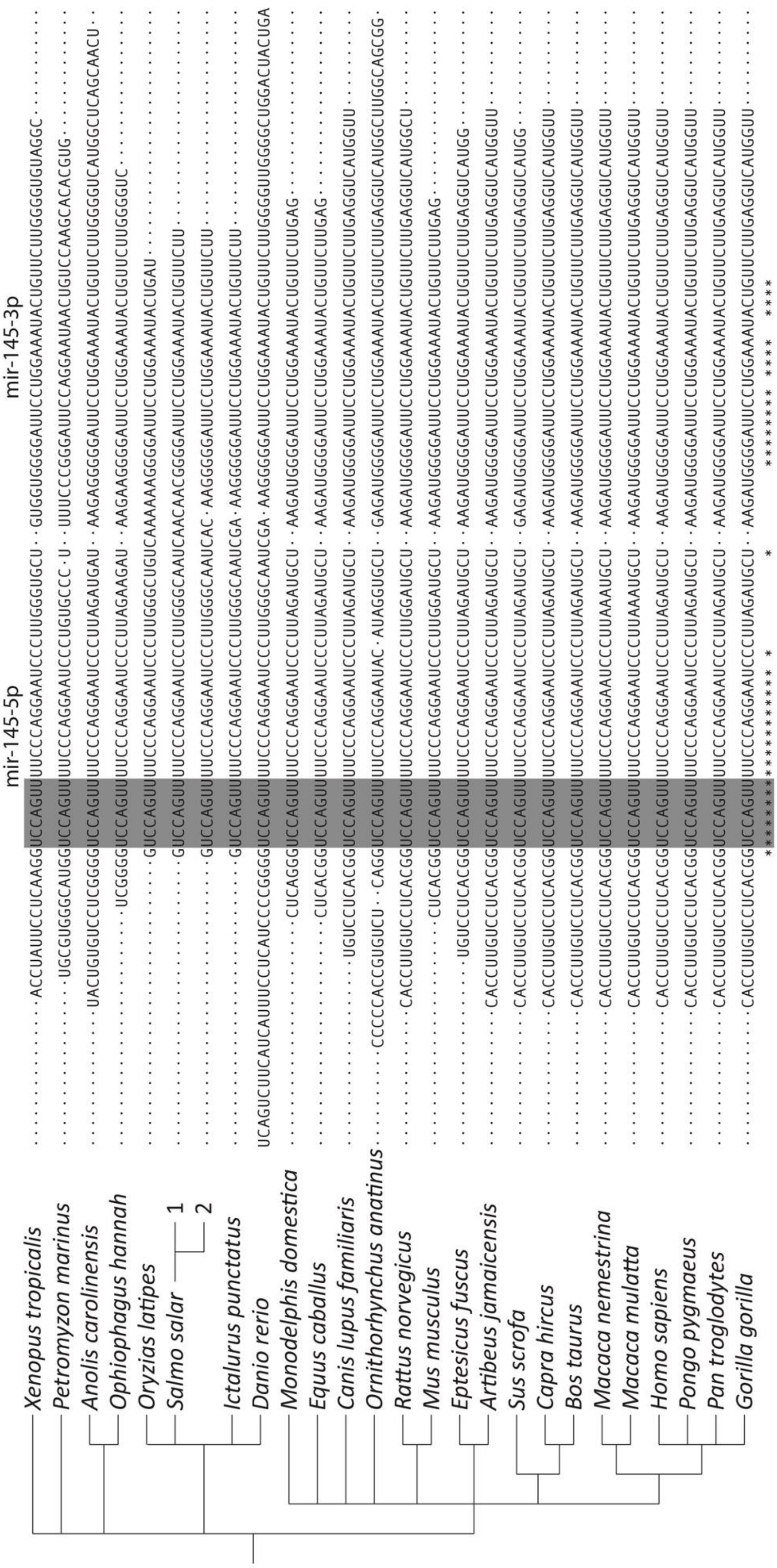

Figure S2. *Conservation of miR-145 across vertebrate species.* Sequence alignment of pre-miR-145 of 25 vertebrate species using Clustal Omega, with taxonomic tree on the left and hairpin sequence on the right. Asterisks represent 100% conserved bases and enclosed in gray rectangles are the seed sequences for miR145-5p.

S3

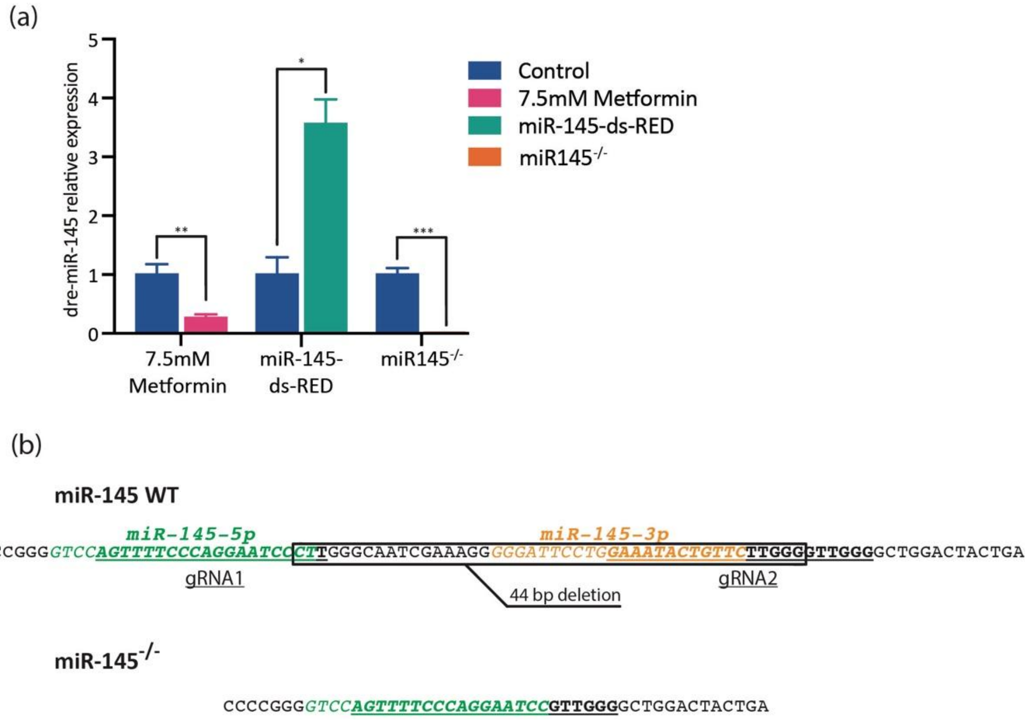

Figure S3. *Measurement of miR-145 levels in 24 hpf treated embryos*. A: Effects of metformin treatment and miR-145-dsRED microinjection on miR-145 expression levels. (±SEM, n=3, two-tailed t-Student test, *P=0.0026, **P=0.0006, ***P<0.0001). B: Schematic representation of miR-145 genomic wild-type sequence (top) and CRISPR/Cas9 mutant sequence (bottom). Mature mir-145-5p and -3p in green and orange respectively, gRNA sequences underlined.

S4

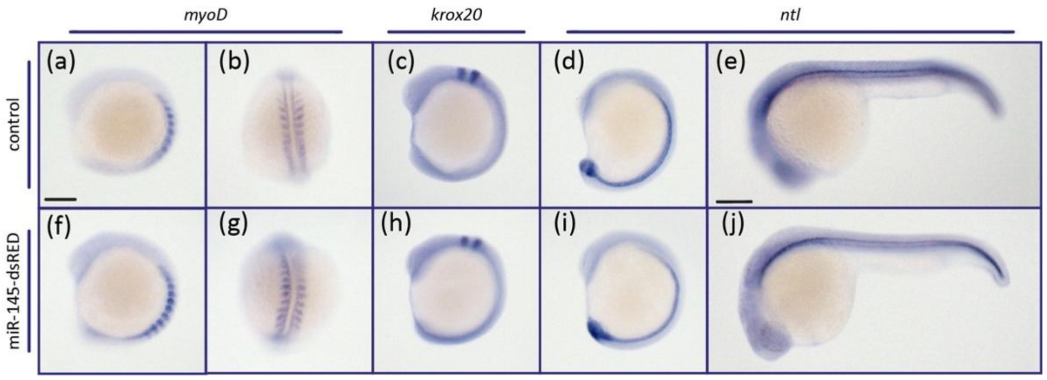

Figure S4. *Effects of miR-145 in the expression of different marker genes*. Whole-mount *in situ* hybridization of *myoD* (**a**, **b**, **f**, **g**), *krox20* (**c**, **h**), and *ntl* (**d**, **i**) performed on 13 hpf miR-145-dsRED or control-dsRED microinjected embryos. Lateral views are dorsal to right (**a**, **c**, **d**, **f**, **h**, **i**); dorsal views are anterior to top (**b**, **g**). Whole-mount *in situ* hybridization of *ntl* in control-dsRED (**e**) or MIR-145-dsRED (**j**) microinjected specimens at 24 hpf, lateral views are head to left. Scale bar 200 μm in (**a**) for (**a**-**d**, **f**-**i**) and in (**e**) for (**e**, **j**).
